# Structure and evaluation of a residency research program in a university hospital

**DOI:** 10.1186/s12909-019-1858-6

**Published:** 2019-11-06

**Authors:** Hani Tamim, Salah Zeineldine, Faysal Tabbara, Samia Khoury, Zeina Akiki, Sara Khansa, Ali Taher

**Affiliations:** 1 Department of Internal Medicine, American University of Beirut Medical Center, Beirut, Lebanon; 2 Clinical Research Institute, American University of Beirut Medical Center, Beirut, Lebanon; 3 Emergency Department, American University of Beirut Medical Center, Beirut, Lebanon; 4 Ann Romney Center for Neurologic Diseases, Brigham and Women’s Hospital and Harvard Medical School, 77 Avenue Louis Pasteur, Boston, MA 02115 USA; 5 Abu Haidar Neuroscience Institute, American University of Beirut Medical Center, Beirut, Lebanon

**Keywords:** Residency research program, University hospital, Academic career, Residents, Structured program

## Abstract

**Background:**

Most academic medical institutions lack a structured program that provides residents with an in-depth research training. The objectives of this paper are to describe a comprehensive residency research program at a university hospital, and to assess the pre- post-self-assessment of research capabilities of resident for the evaluation of the program.

**Methods:**

The residency research program (RRP) was implemented in 2011 as an essential component of the residency program at the American University of Beirut Medical Center. Categorical residents are required to carry out a research project and go through all the steps of the research process from identifying a topic to writing a manuscript. As for evaluating the program, data were collected from residents who graduated between 2014 and 2016 using a questionnaire, which included the overall evaluation of the program, self-assessment on research-related tasks pre- and post- joining the program, as well as general recommendations. The mean scores on the five-point Likert scale were transformed into percentages (0–100%). The average was calculated and the difference in the means was reported.

**Results:**

Overall, 103 residents from the different clinical departments were included in this study. Residents’ self-assessment showed a 19.3% improvement in research-related tasks before and after completion of the RRP (*P* < 0.0001). Most of the residents have either published or are in the process of publishing their projects (34 and 55.3%, respectively). Time management was the most reported challenge. Generally, the program was evaluated positively.

**Conclusion:**

The RRP is a unique, well-structured program, encompassing residents from various clinical departments, which enhances residents’ research capabilities.

## Background

Research is a systematic investigation founded on observation, experimentation and evaluation designed to develop or contribute valid and reliable knowledge [1]. Medical research in particular, whether basic, clinical or epidemiological has enriched and supported the development of the scientific literature worldwide [2]. Early exposure to research experience in undergraduate college and medical students was considered an important opportunity to foster one’s academic medical career [3, 4].

Nonetheless, early exposure to research experience during the residency period is equally beneficial. Actually, research was found to be important in guiding treatment decision and enhanced the residents’ ability to care for patients [5, 6]. Indeed, the number of residents’ scholarly publications was found in a study to be significantly associated with residents’ clinical performance [7]. Investing substantial time and efforts in research allows the practicing resident to keep up with the constantly evolving field of medicine, to comprehend new discoveries and to translate them into patient care [8]. Moreover, getting involved in research prepares residents to become lifelong self-learners and develop their appreciation for research advancement in general [5]. This was confirmed by a longitudinal study of an integrated research residency training curriculum which was found successful in promoting the entry of residents into primary research careers [9]. Furthermore, this period is thought to be a critical juncture for residents who are on their way to start their individual academic path [10]. In addition, residents’ research attitude was found to influence their career expectation and increased their interest in pursuing fellowship training opportunities in their specialty of interest [11].

Acknowledging the aforementioned benefits, several academic institutions have established clinical research as part of their residency-training curriculum. The effectiveness of such programs has been published in a variety of settings and research in this domain has been increasingly discussed [5–9, 12]. However, to our knowledge, existing research residency programs worldwide, did not encompass all the clinical departments and where confined to only a single department within an institution [9, 13]. Moreover, we did not find articles describing a structured research program targeting residents during their training.

The two objectives of this paper are first to describe a comprehensive Residency Research Program (RRP), implemented at a university hospital, and second to perform a pre- and post- self assessment of research capabilities of the residents taking part of the program for the overall evaluation of the program.

## Methods

### Residency research program description

This section covers the first objective of the paper, which is the description of the RRP:

#### RRP

The RRP is a program that was established in 2011 in the department of Internal Medicine at the American University of Beirut (AUBMC), Lebanon, then expanded in 2012 to become an essential requirement of the residency program at all departments of the Faculty of Medicine. Categorical residents are required to carry out a research project throughout their residency program.

#### Eligibility and duration

The RRP is designed to accommodate residents with or without any previous research background. No pre-requisites are needed to be involved in the program. Residents are required to have a serious commitment to fulfill the program requirements. The duration of the RRP is three years, although some specific residency programs might extend beyond that duration (e.g. surgery). Moreover, the program accommodates residents who submit their work prior to the deadlines.

#### RRP process

Following are the different steps of the RRP process.

##### The questionnaires

Two questionnaires, the “resident’s questionnaire” and the “advisor’s questionnaire” need to be filled at the initiation of the process to gather information about both the residents and the advisors. The “resident’s questionnaire” includes information about the resident, their research background, as well as personal assessment of their research skills and knowledge in which the residents had to fill. Similarly, the “advisor’s questionnaire” includes the advisor’s information, research field, interests and ongoing research project filled by the advisor.

##### Resident / advisor matching

The matching process involves mutual agreement of the resident and advisor to work on a specific research project. This matching is done either through self-matching or through the RRP departmental committee. During self-matching, resident identifies the advisor for the specific research question in mind based on similar interests and personal communication. For residents who fail to identify a potential match, the RRP departmental committee carries out the matching based on mutual interest and agreement.

##### Research topic

The research topic could be resident-initiated, where the residents identify the research topic, or faculty-initiated in which the faculty member is the one who provides a project for the resident to work on, which is mainly appropriate to residents who do not have a specific research question in mind.

##### Letter of intent

The resident prepares a letter of intent, which includes information on the resident and the advisor, and a brief description about the project (such as title, objectives, methodology and significance). This letter is submitted and evaluated by the RRP departmental committee.

##### Research proposal

In this step, the resident writes the full proposal, which includes information about the title, starting date, duration of the project, type of project, abstract, objectives, background and significance, research design and methods (including study area, study subjects, study design, sample size, sampling technique, data collection methods, data management and analysis plan), and references. The proposal is submitted to the RRP departmental committee for approval. Moreover, the proposal is submitted to the Institutional Review Board (IRB) at AUB to get the ethical approval.

##### Conducting the research project

After getting the approval on the proposal from both the RRP and the IRB, the resident starts working either on the already existing data (if applicable) or carrying out the data collection and performing data analysis, results interpretation and comparison to literature.

##### Progress presentations

Residents provide an update on the status of their projects, as well as any challenges, by delivering two progress presentations in the presence of members of the departmental committee who provide feedback to the residents.

##### Final report

A final report in the form of a manuscript needs is prepared by the resident, and it includes the following sections: the residents’ and advisors’ information, title, abstract, introduction/background, methodology, results, discussion, conclusion/ recommendations, references, tables and graphs. The report is evaluated by the RRP departmental committee.

##### Final presentation

The resident delivers a final presentation during an RRP research day (see related section). The following parts are presented: title, introduction, objective(s), methods, results, discussion, conclusion, references. The final presentations are evaluated by jury members from different departments.

#### RRP key players

The success of the RRP depends on four key players: the resident, the advisor, the RRP departmental committees, and the RRP committee.

##### Resident

The resident has the major responsibility to carry out the research project in a satisfactory manner. The resident is highly encouraged to submit the prepared report for publications in peer-reviewed journals, as well as to present their research in national and international conferences.

##### Advisor

Any faculty member at the AUBMC could be eligible to be an advisor to one or more residents at any given time. The major responsibility of the advisor is to guide and supervise the resident in the research process. The advisor has to ensure that the resident is carrying out the RRP in a satisfactory manner, through holding regular meetings.

##### RRP departmental committees

The RRP departmental committee includes faculty members who have research expertise in different fields in their respective department. These committees are responsible for carrying out the matching process, evaluating the residents’ work (letter of intent, full proposal, progress presentations, and final report).

##### RRP committee

The RRP committee includes representatives from all the clinical departments, and are responsible for ensuring the efficient flow of the RRP. Members of the committee meet on regular basis to oversee the progress of the program, as well as to address different arising issues.

#### RRP website, educational sessions and portal

A website for the RRP has been developed which includes program information and guide (http://www.aub.edu.lb/fm/medicalresearch/ClinicalandTranslationalResearch/FRRP/Pages/FRRPHome.aspx). Specific forms have been developed and posted online for each of the different steps of the research process, such as the questionnaires, letter of intent, proposal, final report, and presentation templates assessment sheets. These documents were inspired from international guidelines for manuscript writing with the specific structure and word count included in these forms. As for the assessment sheets, it included ratings about the scientific value of the project, as well as the technical and presentation skills of the resident. Moreover, a series of educational sessions on different research topics were recorded and posted online for the residents, which is continuously updated. Residents were continuously encouraged to either attend these sessions or refer to them online. Finally, an online portal has been established, whereby residents can login, create their RRP profile, then fill and submit all the needed documents online.

#### RRP outcome: RRP research day

In recognition to the efforts carried out by the residents, advisors, RRP committee and RRP departmental committee members, an RRP research day is scheduled yearly before the residents’ graduation. During this activity, residents present their work either as poster or oral presentations in front of an audience from the medical field. A jury from different departments evaluate the residents’ work and prizes are awarded to the best presentations.

#### Evaluation of the program

To evaluate the program, questionnaires are filled out by the residents during the research day. The content of the questionnaire will be discussed in the following section.

### Residency research program evaluation

The following section covers the second objective of this paper, which is the evaluation of the RRP.

#### Data collection and measures

Since the program’s establishment, three groups of graduates have successfully completed the RRP process, graduates of years 2014, 2015, and 2016, thus have joined the program in 2011, 2012 and 2013, respectively. Two sources of data were used to obtain an overall evaluation of the program both obtained by giving a self-filling questionnaire to the residents. The first source was the data collected from the residents through their “resident’s questionnaire” filled up upon joining the program (pre-), which included the following: 1- demographic information, 2- residents’ research background before enrolling in the RRP, 3- residents’ self-assessment of their research skills and knowledge before joining the RRP (results presented in Table 2). The second source of data was a similar questionnaire, upon completion of the program (post-), and included the following: 1- demographic information, 2- level of agreement regarding problems faced during the RRP process (results presented in Table 3), 3- advisors’ evaluation, and 4- overall satisfaction and recommendations. Answers to the above questions were mainly based on a 5-point Likert scale.

#### Statistical analyses

The IBM’s Statistical Package for the Social Sciences (SPSS) version 22.0 (IBM, Inc., Chicago, IL) was used for data management and analyses. The mean scores on the 5-point Likert scale were transformed into percentages with a score of 0 corresponding to 0% and a score of 5 corresponding to 100%. Difference between the self-assessment scores in the pre- and post- RRP were also calculated. Data were summarized as numbers and percent for categorical variables and mean and standard deviation for continuous ones. The association between the pre- and post- RRP was assessed by the Chi square test for categorical variables, and Student’s t-test for continuous ones. Statistical significance was indicated at the 0.05% level.

## Results

### Residents’ characteristics

Table 1 summarizes the characteristics of the residents completing the RRP requirements. Although all residents at the AUBMC were eligible in this study, 103 out of 122 residents (84.4%) from the different departments were included in this analyses, mainly: Internal Medicine, Pediatrics, Family Medicine, Emergency Medicine, Anesthesiology, Neurology, Pathology and Lab Medicine, Obstetrics and Gynecology, Ophthalmology, Dermatology, Diagnostic Radiology and Radiation Oncology. Those who are excluded from the analyses were residents with incomplete information at the time of the analyses. The largest number of residents was from the department of Internal Medicine (30.1%), followed by Pediatrics (17.5%) and Family Medicine (11.7%). Moreover, 74.5% had some theoretical background in research, thus previously attended courses on research, statistics, research database experience or evidence base medicine. As for the residents’ previous research experience, 61.2% have been involved prior to their enrollment in the RRP in some stages of the research process, out of whom 14.3% were previously engaged in more advanced stages such as statistical analysis and writing the manuscript.

**Table 1:** Residents’ Characteristics classified by year of graduation, department and previous theoretical background

	*N* = 103 *N* (%)
Year of graduation	
2014	14 (13.6)
2015	42 (40.8)
2016	47 (45.6)
Department	
Internal Medicine	31 (30.1)
Pediatrics	18 (17.5)
Family Medicine	12 (11.7)
Emergency Medicine	11 (10.7)
Anesthesiology	5 (4.9)
Neurology	5 (4.9)
Pathology and Lab Medicine	5 (4.9)
Obstetrics & Gynecology	4 (3.9)
Ophthalmology	4 (3.9)
Dermatology	4 (3.9)
Diagnostic Radiology	3 (2.9)
Radiation Oncology	1 (1.0)
Theoretical Background	
Any theoretical background	76 (74.5)
Research courses	32 (31.4)
Statistic courses	69 (67.6)
Research databases	20 (19.8)
Evidence based medicine	44 (43.6)
Others	7 (6.8)
Involvement in research before RRP	63 (61.2)
Involved in advanced stages (statistical analysis, manuscript write-up)	9 (14.3)

*RRP* Residency Research Program

### Residents’ assessment

Table 2 summarizes residents’ self-assessment according to four different categories pre- and post- completing the RRP. In “Literature Review” category, using PubMed was found to be the variable that had mostly improved with a mean difference of 14.9% (SD = 14.8). In the second category “Writing the Proposal”, applying to the IRB was the variable that has shown the most significant improvement with a mean difference of 29.3% (SD = 22.0) followed by writing the proposal with a mean difference of 26.5% (SD = 22). As for the third category “Data Management and Analyses”, data entry was the variable that has significantly improved with a mean difference of 22.7% (SD = 23.2), followed by data analysis with a mean difference of 21% (SD = 21.1). Finally in the fourth category “Writing”, writing the methods has shown a significant improvement among participants with a mean difference of 21.8% (SD = 19.9). Combining all four categories together, a significant improvement was noted in the various variables with a total mean difference of 19.3% (SD = 14.1). The residents’ self-assessment post completion of the RRP was significant for each individual category and for all categories.

**Table 2:** Residents’ self-assessment of research related tasks before and after completing the RRP, as well as the difference in their assessment

Variables	Pre Mean ± SD	Post Mean ± SD	Diff (Post-Pre) Mean ± SD	*P*-value
Total score 1-Literature Review	66.4 ± 19.5	80.5 ± 15.5	14.1 ± 12.6	< 0.0001
Literature review	65.4 ± 21.5	78.4 ± 17.6	13.3 ± 16.1	< 0.0001
Using PubMed	65.1 ± 20.8	80.0 ± 16.9	14.9 ± 14.8	< 0.0001
Reading articles	70.4 ± 19.9	83.3 ± 15.7	12.9 ± 14.5	< 0.0001
Summarizing articles	65.4 ± 23.2	80.2 ± 17.7	14.7 ± 16.9	< 0.0001
Total score 2-Writing the Proposal	48.0 ± 20.5	71.1 ± 15.8	23.1 ± 17.7	< 0.0001
Defining research objectives	57.8 ± 23.8	76.8 ± 17.1	19.0 ± 18.6	< 0.0001
Constructing questionnaire	49.5 ± 22.9	69.3 ± 17.9	19.8 ± 18.6	< 0.0001
Writing proposal	45.7 ± 24.9	72.2 ± 17.4	26.5 ± 22.0	< 0.0001
Applying to IRB	68.5 ± 21.0	52.6 ± 21.9	29.3 ± 22.0	< 0.0001
Total score 3-Data management and analyses	52.6 ± 21.9	72.8 ± 16.1	20.2 ± 17.7	< 0.0001
Data collection	59.4 ± 26.3	79.4 ± 17.9	20.0 ± 20.0	< 0.0001
Data entry	54.9 ± 27.1	77.7 ± 18.3	22.7 ± 23.2	< 0.0001
Data analyses	45.4 ± 23.5	66.3 ± 20.6	21.0 ± 21.1	< 0.0001
Data summarization	52.3 ± 24.5	71.7 ± 18.6	19.4 ± 21.6	< 0.0001
Tables graphs	52.1 ± 23.4	72.1 ± 17.8	20.0 ± 20.6	< 0.0001
Total score 4-Writing	52.8 ± 21.9	72.2 ± 17.7	19.4 ± 17.1	< 0.0001
Writing introduction	57.4 ± 25.2	77.0 ± 17.2	19.6 ± 20.2	< 0.0001
Writing methods	52.6 ± 25.1	74.4 ± 18.7	21.8 ± 19.9	< 0.0001
Writing results	53.4 ± 24.1	74.4 ± 18.2	21.0 ± 19.2	< 0.0001
Writing discussion	53.3 ± 23.5	71.5 ± 18.2	18.2 ± 17.9	< 0.0001
References	57.4 ± 26.3	76.2 ± 18.6	18.8 ± 21.4	< 0.0001
Submission publication	39.4 ± 24.1	59.8 ± 25.6	20.4 ± 23.3	< 0.0001
Study presentation	60.4 ± 25.6	78.8 ± 18.6	18.4 ± 21.8	< 0.0001
Total score	54.5 ± 19.5	73.8 ± 15.0	19.3 ± 14.1	< 0.0001

*IRB* Institutional Review Board, *SD* standard deviation; *RRP* Residency Research Program

### Residents’ publications after enrollment in the RRP

**Fig. 1:**
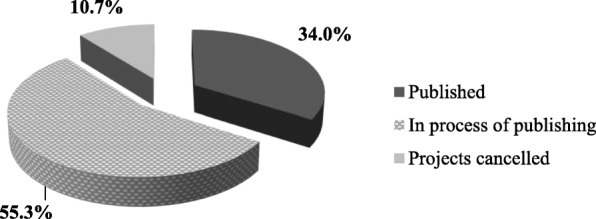
Residents’ publications’ distribution after enrollment in the RRP (*n* = 36)

As for the publication rate after RRP completion, results are reported in Fig. 1. More specifically, 34% have published their RRP studies, 55.3% are in the process (writing or submitting the manuscript for publication), whereas, 10.7% have decided not to submit their work to journals for publication due to various reasons (e.g. the results were not conclusive, missing data …).

### Problems faced by the residents during the RRP

Table 3 provides data regarding problems faced by residents during the RRP at various levels, such as time issues, data processing, logistics and many others. Time management was noted to be a significant barrier for residents where 59.6% (SD = 26.5) of the residents reported time management issues. Moreover, 53.9% (SD = 23.4) had some problems in the data analysis. Similarly, 53.2% (SD = 23.7) of the residents had difficulties identifying a topic for their research project and 51.1% (SD = 24.1) had some delays in getting IRB approval for their research project.

**Table 3:** Distribution of problems faced by residents during the RRP, stratified by the different phases of the research project

Problems faced by residents during the RRP	N = 103 Mean ± SD
Total score 1-Time issues	57.2 ± 24.5
Time management	59.6 ± 26.5
Time frame for the project	55.1 ± 25.2
Total score 2-Data processing	50.4 ± 18.8
Data analyses	53.9 ± 23.4
Data collection	51.7 ± 24.3
Data summarization	49.5 ± 20.7
F Data entry	46.9 ± 21.9
Total score 3-Logistics	50.1 ± 17.1
Topic Identification	53.2 ± 23.7
Proposal Writing	49.5 ± 20.9
Report writing	47.9 ± 18.9
Total score 4-Other	45.3 ± 16.1
Getting approval from IRB	51.1 ± 24.1
Lack of knowledge	48.4 ± 22.4
Identifying a supervisor	44.6 ± 22.4
Supervisor relationship	37.5 ± 18.4
Total Score	49.7 ± 16.4

*RRP* Residency Research Program

### Residents’ evaluation and feedback

The overall program evaluation score by the residents’ was found to be 73.0% (SD = 12.6) (Table 4). On a 5-point Likert scale, the highest evaluation criteria were that “residents will carry out research in the future” with a score of 78.8 (SD = 15.0). Out of the several recommendations, 89.2% of residents’ recommended “more teaching in data analyses”, 83.2% recommended “more teaching in paper writing”, and 73.5% recommended “allocated a dedicated time for RRP”. Moreover, residents’ feedback on RRP advisors yielded a total average score of 90.8% (SD = 16.0), where the mean scores ranged between 88.4% for “allocated enough time” to 91.8% for “being helpful during the process” (Table 5).

**Table 4:** Residents’ evaluation and recommendation of the program

RRP Evaluation and recommendations	*N* = 103
Evaluation	Mean ± SD
I will carry out research in the future	78.8 ± 15.0
The FRRP is an important component of the curriculum	77.1 ± 20.1
I have the expertise to initiate a research project	76.3 ± 16.3
The FRRP was not a waste of my time	73.2 ± 22.5
I have the expertise to finalize a research project	72.8 ± 17.7
I have the expertise to present in national and international conferences	72.8 ± 19.6
The FRRP enhanced my interest in research	71.3 ± 22.0
The time allocated for the FRRP could not have been utilized for better purposes	70.1 ± 23.1
I have the expertise to publish in medical journals	65.3 ± 21.6
Total Score	73.0 ± 12.6
Recommendations	N (%)
More teaching in data analysis is required	91 (89.2)
More teaching in paper writing is required	84 (83.2)
A dedicated time needs to be given when joining the RRP	75 (73.5)
More time needs to be given to do the RRP project	62 (60.8)
Supervisors needs to be more aware and committed to the projects	54 (52.9)
RRP kept as it is	43 (42.6)
RRP made an optional part of the curriculum	39 (38.6)
RRP cancelled from the curriculum	9 (8.9)

RRP: Residency Research Program

**Table 5:** Residents’ feedback on RRP advisors

Residents’ feedback on RRP advisors	N = 103 Mean ± SD
Was helpful during the process	91.8 ± 16.4
Reviewed documents promptly	91.5 ± 16.5
Had adequate research expertise	91.5 ± 16.6
Provided important advice	91.3 ± 16.3
Supported me	91.3 ± 16.5
Provided constructive feedback	90.7 ± 17.5
Was easy to be reached	89.7 ± 17.9
Allocated enough time	88.4 ± 18.5
Total Score	90.8 ± 16.0

*RRP* Residency Research Program

## Discussion

This paper provides a comprehensive description of a research program targeting categorical medical residents (RRP) at the AUBMC, as well as a pre- and post- self-assessment of the program. The RRP is one of the essential components of the residency curriculum at the Faculty of Medicine. This program enabled residents to develop a deeper understanding of the scientific and evidence based medicine (EBM) which is beyond what is offered in the core curriculum as shown by previous similar research [14]. Residents positively evaluated the RRP and showed a significant improvement in several research areas.

The RRP is a well-structured program, which follows an organized process starting with topic selection to manuscript finalization. The program is unique as it is the only residency research program, to our knowledge, to encompass residents from the various clinical departments, while previous research has only focused on single departments at a time [9, 13]. Moreover, our program establishes a structured timetable for residents, as it was lacking in similar programs described in the literature [15, 16]. As lacking in most other similar programs, an important aspect in the RRP is the development of educational sessions covering basic to advanced topics in research methodology [17, 18]. Resident’s research background and experience was collected at the beginning of the program, which was not addressed in other similar programs [13, 16, 19]. This led to tailoring these educational sessions for the residents which were posted on the university website to be accessed by the residents. This step was beneficial for them in conducting their projects as this was lacking in other research programs implemented in other institutions.

The RRP has subjectively assessed the residents’ knowledge and experience in research pre- and post- the completion of the program. More specifically, residents enhanced their knowledge and expertise pertaining to the different steps of the research process. The program enabled the residents to publish their articles in peer-reviewed journals, as well as to present in international conferences. This finding is in line with previous literature as it has been found that a dedicated research program during residency is indeed linked to a greater number of publications by residents [20]. Residents, throughout the process have also improved their communication skills while preparing their oral presentations as was outlined in previous similar studies [21]. In addition, residents were given the chance to get connected with advisors from various backgrounds with different specialty and subspecialty areas. Indeed having a well-structured relation between the advisor and the resident is crucial for the success of the any residency research program [16]. Indeed, the RRP was considered a great start for residents who wish to pursue a research career, along to their clinical practice, as previously reported [3, 4].

An additional aspect of the uniqueness of this program is the inclusion of residents’ self-assessment, as well as an overall evaluation of the program. Moreover, residents offered recommendations for future improvement. In fact, continuous evaluation of any newly established program, whether in medicine or any other discipline, leads to its rapid growth and its improvement [22]. Such an evaluation has not been reported in papers describing similar programs, except for a single paper only reporting the number of publications and residents’ enrollment in a postdoctoral research fellowship [9].

The RRP was equally beneficial to the advisors who got support from the residents in their ongoing research projects or any new research within the advisors’ research interests. Indeed, research as a requirement during residency was found to enhance collaborative resident and faculty publication productivity [19]. As for the institutional level, this program has increased the number of ongoing research projects and publications in the various clinical departments and has allowed cross-departmental collaboration. More specifically, several topics were tackled through collaboration between different clinical departments. Finally, the RRP has established a research culture among the members of the AUBMC community.

Like any other residency research program implemented in an institution, the RRP has faced several challenges at different levels, such as financial, administrative, and time-related issues. Financial barriers were among the challenges of our program, more specially the limited financial support allocated for residents’ research projects, which could be addressed by encouraging residents to apply for internal and external funding, as well as to secure institutional budget allocated for residents’ research. Moreover, the advisor did not explicitly assess the research skills of the residents, which should be tackled in future implementation of this program. Another challenge was the administrative issues that needed to be worked out to ensure a proper structure and flow of the program. Moreover, facing some delays at different stages of the research process, one of which was securing the IRB approval, as most of these projects were new projects was another challenge. Another main challenge was pertaining to time-related issues, mainly the commitment to the RRP deadlines from the committee members, advisors and residents. Unfortunately, as reported previously [15], residents have complained about the lack of protected time in their schedule for undergoing research and found some difficulties managing their clinical work and practice with the research deadlines required by the RRP. For the purpose of overcoming these challenges, time tables were developed taking into consideration the expected delays at early stages. Another strategy to overcome time-related issues was to send e-mails to remind residents, advisors and committee members of the RRP deadlines, to schedule meetings with representatives to discuss the residents’ progress, schedule progress presentations with residents to discuss challenges and ways to overcome them and to offer assistance in the different stages of their research projects. Another approach to deal with this challenge is to introduce workshops on time management skills offered to residents with the aim to reach a well-balanced clinical/research time allocation. Finally, although the RRP is a well-structured and positively evaluated program, replicating it in other institutions whether in Lebanon or in the surrounding countries might be faced with some difficulties depending on the institution’s mission and regulations.

## Conclusion

The RRP is a mandatory research program that provides residents at AUBMC with the opportunity to conduct research in different areas under the supervision of advisors in different specialties. This program has created a unique research culture allowing residents to foster their academic medical career, thus becoming better clinicians and researchers. The RRP is a unique, well-structured program, encompassing residents from various clinical departments, as well as collecting feedback and evaluation on regular basis. Based on continuous evaluation, we anticipate more changes to be implemented with the hope to reach a program with even higher standards. We envision expanding it in the subsequent years to include clinical fellows at AUBMC and probably to expand and collaborate with other academic medical schools at the national and international levels. Future papers describing similar programs will have an added contribution to enhancing residents’ involvement in research.

## Data Availability

The datasets used and/or analysed during the current study are available from the corresponding author on reasonable request.

## Abbreviations

AUBMC: American University of Beirut Medical Center
EBM: Evidence based medicine
IRB: Institutional Review Board
RRP: Residency Research Program
